# Consumption of Dairy Yogurt Containing *Lactobacillus paracasei* ssp. *paracasei*, *Bifidobacterium animalis* ssp. *lactis* and Heat-Treated *Lactobacillus plantarum* Improves Immune Function Including Natural Killer Cell Activity

**DOI:** 10.3390/nu9060558

**Published:** 2017-05-31

**Authors:** Ayoung Lee, Young Ju Lee, Hye Jin Yoo, Minkyung Kim, Yeeun Chang, Dong Seog Lee, Jong Ho Lee

**Affiliations:** 1 National Leading Research Laboratory of Clinical Nutrigenetics/Nutrigenomics, Department of Food and Nutrition, College of Human Ecology, Yonsei University, Seoul 03722, Korea; ao0511@naver.com (A.L.); juny9558@naver.com (Y.J.L.); hyejin10432@hanmail.net (H.J.Y.); jyee9311@gmail.com (Y.C.); 2 Department of Food and Nutrition, Brain Korea 21 PLUS Project, College of Human Ecology, Yonsei University, Seoul 03722, Korea; 3 Research Center for Silver Science, Institute of Symbiotic Life-TECH, Yonsei University, Seoul 03722, Korea; mkkim0106@yonsei.ac.kr; 4 Purmil Co., Ltd., Seoul 07281, Korea; dslee@purmil.co.kr

**Keywords:** immune function, IFN-γ, NK cell activity, probiotics

## Abstract

The aim of this study was to investigate the impact of consuming dairy yogurt containing *Lactobacillus paracasei* ssp. *paracasei* (*L. paracasei*), *Bifidobacterium animalis* ssp. *lactis* (*B. lactis*) and heat-treated *Lactobacillus plantarum* (*L. plantarum*) on immune function. A randomized, open-label, placebo-controlled study was conducted on 200 nondiabetic subjects. Over a twelve-week period, the test group consumed dairy yogurt containing probiotics each day, whereas the placebo group consumed milk. Natural killer (NK) cell activity, interleukin (IL)-12 and immunoglobulin (Ig) G1 levels were significantly increased in the test group at twelve weeks compared to baseline. Additionally, the test group had significantly greater increases in serum NK cell activity and interferon (IFN)-γ and IgG1 than placebo group. Daily consumption of dairy yogurt containing *L. paracasei*, *B. lactis* and heat-treated *L. plantarum* could be an effective option to improve immune function by enhancing NK cell function and IFN-γ concentration (ClinicalTrials.gov: NCT03051425).

## 1. Introduction

The population of individuals above the age of 60 is steadily increasing in Korea. Elderly individuals suffer from more frequent and more severe infections than younger individuals for reasons including epidemiological factors, immunosenescence and malnutrition as well as various age-associated physiological alterations [1]. Human immune function also undergoes adverse changes with aging, including immune senescence, which potentially increases the risk of certain infections and cancers [2,3].

Consumption of yogurt could improve immune function based on its composition of probiotics, zinc, vitamin B6, and protein, which are associated with immune enhancement [4,5]. Among these components, probiotics are regarded as the most important in terms of stimulating the immune system [6]. Many studies have demonstrated that intake of some probiotic strains can affect the immune response with different manifestations [7,8,9,10]. For example, Rizzardini et al. [10] showed that supplementation with *Bifidobacterium animalis* ssp. *lactis* (BB-12^®^) and *Lactobacillus paracasei* ssp. *paracasei* (L. casei 431^®^) could be an effective means to improve immune function by augmenting the systemic immune response to challenge using a vaccination model in healthy subjects. Makino et al. [9] showed that consumption of yogurt fermented with *Lactobacillus delbrueckii* ssp. *bulgaricus* OLL1073R-1 augmented natural killer (NK)-cell activity and reduced the risk of infection in elderly individuals. Additionally, Kawashima et al. [8] observed that *Lactobacillus plantarum* (*L. plantarum*) strain YU has a beneficial effect in activating helper T lymphocyte (Th)-1 immune responses and preventing viral infection.

Interestingly, some studies of heat-treated or heat-killed *Lactobacillus* have also shown a relationship with enhanced immunity. Tobita et al. [11] reported that heat-treated *Lactobacillus crispatus* KT strains could modulate the type 1/type 2 Th cell balance, reducing allergic symptoms in mice. Murosaki et al. [12] revealed that heat-killed *L. plantarum* L-137 might augment immunity in response to increased plasma levels of interleukin (IL)-12, previously known as natural killer cell stimulatory factor, which were obtained in mice treated with this strain. Hirose et al. [13] also found that heat-killed *L. plantarum* L-137 positively influenced acquired immune responses in healthy adults. Moreover, Lee et al. [14] reported that dead nano-sized *L. plantarum* reduced the expression of inflammatory markers in mouse colonic tissues.

Therefore, the aim of this study was to investigate the impact of consuming dairy yogurt containing *Lactobacillus paracasei* ssp. *paracasei* (*L. paracasei*), *Bifidobacterium animalis* ssp. *lactis* (*B. lactis*) and heat-treated *L. plantarum* on NK cell activity and circulating levels of cytokines and immunoglobulin (Ig) in elderly individuals (≥60 years).

## 2. Materials and Methods

### 2.1. Study Subjects

The study included 200 nondiabetic (fasting serum glucose concentration <126 mg/dL) subjects over 60 years in age with white blood cell levels between 4 × 10^3^/μL and 10 × 10^3^/μL. Participants were recruited from the Goyang-si Heendol Community Welfare Center (Goyang, Korea) via poster advertisements between March 2016 and December 2016. Volunteers who agreed to participate and provided written informed consent were screened to measure white blood cell and fasting serum glucose levels. After the screening, those who met the inclusion criteria were enrolled. The following exclusion criteria were applied: regular consumption (more than 5 times per week) of any probiotic products or taking medicine related to inflammation within one month before screening, history/presence of diabetes, allergy to milk protein, epilepsy, liver disease, kidney disease, immune disease, cancer, or medication/alcohol abuse. Before participation, the purpose of the study was carefully explained to all subjects and written informed consent was obtained. The study protocol was approved by the Institutional Review Board of Yonsei University (1040917-201603-BR-151-06) and was conducted in accordance with the Helsinki Declaration.

### 2.2. Study Design and Intervention

A randomized, open-label, placebo-controlled study was conducted on 200 nondiabetic subjects. Over a twelve-week period, the test group (*n* = 100) consumed one bottle (120 mL) of dairy yogurt containing *L. paracasei* (L. casei 431^®^) at 12.0 × 10^8^ cfu/day, *B. lactis* (BB-12^®^) at 12.0 × 10^8^ cfu/day and 0.0175% heat-treated *L. plantarum* (nF1) once per day. The placebo group (*n* = 100) consumed the same volume of milk once per day (NCT03051425, http://www.clinicaltrials.gov). The two probiotic strains (L. casei 431^®^ and BB-12^®^) and heat-treated *L. plantarum* (nF1) were provided by Chr.Hansen A/S (Hθrsholm, Denmark) and Biogenics Korea Co., Ltd (Seoul, Korea), respectively. The final products, including dairy yogurt and milk, were provided by Purmil Co., Ltd. (Seoul, Korea). Computer-generated block randomization was used (placebo:dairy yogurt = 1:1). The study was divided into two periods: the pre-ingestion period, in which nondiabetic subjects did not ingest test or placebo products for two weeks (from screening to week 0), and the ingestion period, in which subjects ingested dairy yogurt or milk during the twelve-week study (from week 0 to week 12).

### 2.3. Anthropometric Parameters and Blood Pressure

Body weight (in lightweight clothes and without shoes) (UM0703581; Tanita, Tokyo, Japan) and height (GL-150; G-tech International, Uijeongbu, Korea) were measured in the morning, and body mass index (BMI) was calculated in units of kilograms per square meter (kg/m^2^). Anthropometric parameters were assessed at weeks 0 and 12. During each testing session, systolic and diastolic blood pressure (BP) were assessed in the supine position after a resting period (20 min). BP was measured twice on the left arm using an automatic BP monitor (FT-200S; Jawon Medical, Gyeongsan, Korea); the two measurements were then averaged.

### 2.4. Serum Glucose and Lipid Profiles

Serum fasting glucose levels were measured via the hexokinase method; fasting triglyceride and total and low density lipoprotein (LDL) cholesterol levels were measured via enzymatic assays; and high density lipoprotein (HDL) cholesterol levels were measured via selective inhibition. All measurements were taken using a Hitachi 7600 autoanalyzer (Hitachi Ltd., Tokyo, Japan).

### 2.5. Serum Albumin, White Blood Cell, High-Sensitivity C-Reactive Protein and Immunoglobulin G Levels

Serum albumin concentrations were analyzed via the BCG method using an ALB kit (Roche, Basel, Switzerland) with a Hitachi 7600 autoanalyzer (Hitachi Ltd., Tokyo, Japan). White blood cell levels were determined using a HORIBA ABX diagnostic analyzer (HORIBA ABX SAS, Parc Euromedicine, Montpellier, France). Serum high-sensitivity C-reactive protein (hs-CRP) levels were measured via the turbidity method by a latex agglutination immunoassay using a Hitachi 7600 autoanalyzer. The concentrations of serum immunoglobulin G1 and G3 were analyzed via immunoturbidmetric assay using a COBAS Integra 800 (Roche Diagnostics, Rotkreuz, Switzerland).

### 2.6. Cytokine Assays

The serum level of tumor necrosis factor (TNF)-α was measured using a Bio-Plex™ Reagent Kit on a Bio-Plex^TM^ system (Bio-Rad Laboratories, Hercules, CA, USA) according to the manufacturer’s instructions. Interferon (IFN)-γ in the serum was analyzed using an IFN gamma High-Sensitivity Human ELISA Kit (Covalab, Villeurbanne, France) according to the manufacturer’s instructions. IL-12 in the serum was measured using a High-Sensitivity Human IL-12 (P70) ELISA kit (Bosterbio, Pleasanton, CA, USA). The absorbance of the reaction mixtures was read at 450 nm using a Victor^TM^ × 5 Multilabel HTS Reader (PerkinElmer, Waltham, MA, USA).

### 2.7. Isolation of PBMCs

Whole blood was mixed with the same volume of RPMI 1640 (Gibco, Invitrogen Co., Waltham, MA, USA), gently overlaid on Histopaque-1077 (Sigma-Aldrich, St. Louis, MO, USA), and then centrifuged at 1800 rpm for 20 min at 15 °C. After separation, the peripheral blood mononuclear cell (PBMC) layer was isolated, washed twice, and resuspended in RPMI 1640. PBMCs were cultured with streptomycin for NK cell cytotoxicity assays.

### 2.8. Cytotoxic Activity of NK Cells

The cytolytic activity of NK cells was determined using a CytoTox96^®^ Non-radioactive Cytotoxicity Assay Kit (Promega, Madison, WI, USA). To assay NK cell cytotoxic activity, PBMCs isolated from each subject were incubated with K562 cells. Briefly, PBMCs (effector cells, E) were seeded with 2 × 10^4^ K562 cells (target cells, T) per well at ratios of 10:1, 5:1, 2.5:1, 1.25:1 and 0.625:1. The plates were treated at different E:T ratios (10:1, 5:1, 2.5:1, 1.25:1 and 0.625:1) and were incubated at 37 °C with 5% CO_2_ overnight, according to the manufacturer’s instructions. Finally, NK cell activity was measured using a Victor^TM^ × 5 Multilabel Plate Reader (PerkinElmer, Waltham, MA, USA) at 490 nm and was calculated using the following formula:

(1) $$\%\;\mathrm{Cytotoxicity}=\frac{\mathrm{Experimental}-\mathrm{Effector}\;\mathrm{Spontaneous}-\mathrm{Target}\;\mathrm{Spontaneous}}{\mathrm{Target}\;\mathrm{Maximum}-\mathrm{Target}\;\mathrm{Spontaneous}}\times 100$$

### 2.9. Daily Energy Intake and Physical Activity Measurements

Information about the subjects’ usual diets was obtained using both a 24-h recall method and a semi-quantitative food frequency questionnaire. We used the former to carry out our analyses and the latter to check if the data collected by the 24-h recall method were representative of their usual dietary patterns. All subjects were given written and verbal instructions by a registered dietitian on how to complete a three-day (two weekdays and one weekend day) dietary record every six weeks. Dietary energy values and nutrient contents from these 3-day food records were calculated using the Computer Aided Nutritional analysis program (CAN-pro 3.0, Korean Nutrition Society, Seoul, Korea). A standardized 3-day physical activity record was also completed at home on the same days in which the dietary record was completed. Total energy expenditure (kcal/day) was calculated from each subject’s activity patterns, including their basal metabolic rate, physical activity over 24 h, and specific dynamic action of the food consumed. Basal metabolic rates for each subject were calculated using the Harris-Benedict equation.

### 2.10. Statistical Analysis

Statistical analysis was performed using SPSS, version 23.0 (IBM/SPSS, Chicago, IL, USA). Skewed variables were logarithmically transformed. Independent *t*-tests were used to compare parameters between the placebo and test groups. Paired *t*-tests were used to compare parameters between the baseline measurements and those collected at the 12-week follow-up. Pearson’s correlation coefficient was used to examine the relationships between variables. The results are expressed as the mean ± standard error. For descriptive purposes, mean values are presented using untransformed values. A two-tailed *p*-value of less than 0.05 was considered statistically significant.

## 3. Results

### 3.1. Effects on Clinical Characteristics Following Twelve Weeks of Consuming Dairy Yogurt Containing L. paracasei, B. lactis and Heat-Treated L. plantarum

This study initially enrolled 200 subjects: 48 subjects (21 placebo and 27 test subjects) were later omitted, with 23 subjects discontinuing the study for personal reasons and 4 participants requiring antibiotics. Ten participants who had poor compliance (less than 80%) and 4 subjects who experienced weight change greater than 5% from baseline were excluded from the final analysis. Seven subjects were also excluded because their white blood cell level at baseline did not meet the study criteria. Ultimately, 152 subjects (79 placebo and 73 test subjects) were included in the final analysis. No adverse events were reported from the participants. Table 1 shows the clinical characteristics at baseline and at twelve weeks for the placebo and test groups. At baseline, there were no significant differences between the two groups in age, gender distribution, smoking and drinking, BMI, systolic and diastolic BP, serum glucose, lipid profiles, albumin, leukocyte counts, or hs-CRP (Table 1). After 12 weeks of treatment, significant increases were found in serum triglyceride in the test group (yogurt group) and in hs-CRP in the placebo group (milk group). However, there were no significant changes (differences from baseline) in triglyceride or hs-CRP between the placebo and test groups (Table 1). The estimated total calorie intake, physical activity, percent protein intake, percent fat intake, and percent carbohydrate intake did not significantly differ between the two groups at baseline, week six, or week twelve (data not shown).

### 3.2. Effects on Serum Cytokine and Immunoglobulin Concentrations Following Twelve Weeks of Consuming Dairy Yogurt Containing L. paracasei, B. lactis and Heat-Treated L. plantarum

**Figure 1:**
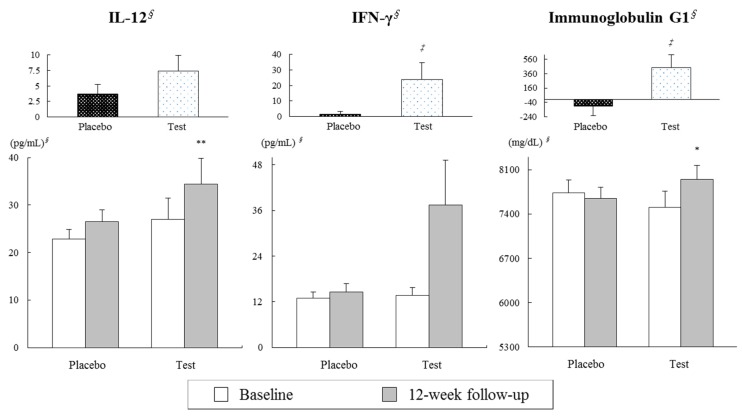
Effects on serum interleukin (IL)-12, interferon (IFN)-γ, and immunoglobulin G1 concentrations following 12 weeks of consuming dairy yogurt containing *L. paracasei*, *B. lactis* and heat-treated *L. plantarum*. Mean ± SE. *^∮^* tested by logarithmic transformation. * p < 0.05 and ** p < 0.01 derived from paired *t*-tests within each group. *^‡^ p* < 0.05 derived from independent *t*-tests at changed value and adjusted for baseline values, sex, and changes in diastolic blood pressure (BP).

No significant differences were found in serum concentrations of TNF-α, IgG3 (Table 1), IL-12, IFN-γ, or IgG1 (Figure 1) at baseline between the placebo and test groups. After twelve weeks of treatment, significant increases were found in IL-12 and IgG1 in the test group. In comparing differences from baseline between the placebo and test groups, the test group exhibited greater increases in serum IFN-γ (*p* = 0.041) and IgG1 (*p* = 0.022) concentrations (Figure 1).

### 3.3. Effects on NK Cell Activity Following Twelve Weeks of Consuming Dairy Yogurt Containing L. paracasei, B. lactis and Heat-Treated L. plantarum

NK cell activity (%) was measured based on E:T ratios of 10:1, 5:1, 2.5:1, 1.25:1 and 0.625:1. As shown in Table 2, no significant differences were found in NK cell activity measured at baseline between the placebo and test groups under any condition. Compared to baseline, NK cell activity significantly increased for all E:T ratios in the test group at twelve weeks, whereas the placebo group showed no significant changes. When we compared the changes between the placebo and test groups, the test group had greater increases in serum NK cell activity at ratios of E:T = 10:1 (*p* < 0.001), E:T = 5:1 (*p* < 0.001), E:T = 2.5:1 (*p* < 0.001), E:T = 1.25:1 (*p* = 0.003) and E:T = 0.625:1 (*p* = 0.002) after adjusting for baseline values, sex, and changes in diastolic BP (Table 2).

## 4. Discussion

The main findings of the present study indicated that daily supplementation of one bottle (120 mL) of dairy yogurt containing *L. paracasei*, *B. lactis* and heat-treated *L. plantarum* led to beneficial immunostimulatory effects in healthy elderly subjects. In comparison with the placebo group, the test group showed significantly greater increases in serum NK cell activity at E:T ratios of 10:1, 5:1, 2.5:1, 1.2.5:1 and 0.625:1 after adjusting for baseline values, sex, and changes in diastolic BP. This finding is important because NK cells play an important role in the innate immune response. Indeed, Kawashima et al. [8] showed that the *L. plantarum* strain YU enhances NK cell activity in spleen cells, and Dallal et al. [7] recently reported that oral administration of *L. casei* significantly increased NK cytotoxicity in spleen cell cultures from mice bearing invasive ductal carcinoma. Takeda et al. [15] demonstrated that habitual intake of a fermented milk drink containing the *L. casei* strain Shirota increased NK cell activity in middle-aged volunteers. Moreover, an enhancement in NK cell activity in mouse spleen cells was also found after oral administration of *L. bulgaricus* OLL1073R-1 or yogurt fermented with this strain [16]. Therefore, the present result showing enhanced NK cell activity in the test group probably resulted from consumption of yogurt containing *L. paracasei*, *B. lactis* and heat-treated *L. plantarum*, which could enhance the immune response, particularly in immunocompromised populations such as the elderly.

NK cells provide a substantial defense against viral infection [17], and low NK cell activity was shown to be associated with the development of infections in healthy elderly subjects [18]. In a murine experiment, NK cells were shown to be a major source of IFN-γ, a potent immune-stimulatory cytokine [19] also known for its antiviral, immunoregulatory, and anti-tumor properties [20]. IFN-γ also has an effect on NK cell regulation [21]; therefore, the increase in serum IFN-γ concentration accompanying the increase in NK cell activity measured in the test group in this study could have contributed to the immune-enhancing action of supplementation with dairy yogurt containing *L. paracasei*, *B. lactis* and heat-treated *L. plantarum*.

IFN-γ is produced not only by NK cells but also T and B cells [22]. Th cells are divided into two functional subclasses, Th1 and Th2, based on the cytokines they produce and their effects on cell-mediated and humoral immunity [23]. Th1 cells produce IL-2, IFN-γ, TNF-α, and IL-12 and enhance cell-mediated immunity, whereas Th2 cells produce IL-4, IL-5, IL-6, and IL-10 and up-regulate humoral immunity. Increased IL-12 production exerts a protective effect, which may be related to increased cellular immunity and phagocytic function [24]. IL-12 is also important for the induction of Th1 immunity [25] and directly activates CD56+ NK cell-mediated cytotoxicity [26]. In the present study, IL-12 levels significantly increased in the test group after twelve weeks of daily consumption of dairy yogurt compared with before supplementation.

The result that IgG1 levels significantly increased after the yogurt supplementation suggests that Th cell activity was promoted. Notably, in addition to being preferentially correlated with Th cells, IgG1 is also associated with optimal activation of complement [27].

The current study had several limitations. First, dietary intake was based on self-reports obtained from weighed food, which could have led to errors. However, measurement errors from self-reported dietary intake and lifestyle variables have been shown to be relatively small [28]. Second, we specifically focused on Korean nondiabetic subjects greater than 60 years in age. Typically, Korean adults consume low amounts of dairy products (60–69 years, 70.1 ± 5.4 g/day) according to the Korea Health Statistics 2013: Korean National Health and Nutrition Examination Survey (KNHANES VI-1). Therefore, our data cannot be generalized to other ethnic groups, other age groups, or severely obese subjects. Finally, we used milk as placebo product. Therefore, we could not conclude the results of this study were derived from probiotics or differences between milk and yogurt. Despite these limitations, this study showed that after consumption of dairy yogurt containing probiotics for 12 weeks, significant increases were found in NK cell activity and serum levels of IL-12 and IgG1. The test group also exhibited greater increases in NK cell activity and serum IFN-γ and IgG1 concentrations than the controls. Importantly, this is the first clinical study to investigate the effect of heat-treated *L. plantarum* (nF1) on improving immune function. The results demonstrated that consumption of yogurt containing *L. paracasei*, *B. lactis* and heat-treated *L. plantarum* could enhance the immune response, particularly in immunocompromised populations such as the elderly.

## 5. Conclusions

In this study, we examined the impact of consuming dairy yogurt containing *L. paracasei*, *B. lactis* and heat-treated *L. plantarum* over a twelve-week period on immune function. Consumption of yogurt containing probiotics increased NK cell activity, IL-12 and IgG1 in the test group, and increases in NK cell activity and IFN-γ and IgG1 in the test group were significantly greater than placebo group. The results suggest that daily consumption of dairy yogurt containing *L. paracasei*, *B. lactis* and heat-treated *L. plantarum* could improve immune function by enhancing NK cell activity.

## Figures and Tables

**Table 1:** Effects on clinical and biochemical characteristics following 12 weeks of consuming dairy yogurt containing *L. paracasei*, *B. lactis* and heat-treated *L. plantarum*.

	Total Subjects (*n* = 152)				*p^a^*	*p^b^*	*p^c^*
	Placebo (*n* = 79)		Test (*n* = 73)				
	Baseline	Follow-up	Baseline	Follow-up			
Age (year)	65.7 ± 0.56		65.7 ± 0.50		0.988		
Male/Female *n*, (%)	24 (30.4)/55 (69.6)		21 (28.8)/52 (71.2)		0.828		
Current smoker *n*, (%)	4 (5.1)		7 (9.6)		0.282		
Current drinker *n*, (%)	32 (40.5)		25 (34.2)		0.426		
BMI (kg/m^2^)	23.7 ± 0.31	23.7 ± 0.33	23.6 ± 0.25	23.7 ± 0.26	0.856	0.895	
Change	−0.03 ± 0.06		0.09 ± 0.06				0.131
Systolic BP (mmHg)	125.7 ± 1.73	123.4 ± 1.72	124.3 ± 1.94	122.9 ± 1.94	0.601	0.832	
Change	−2.23 ± 1.50		−1.42 ± 1.61				0.713
Diastolic BP (mmHg)	77.1 ± 0.93	76.9 ± 1.22	76.8 ± 1.19	75.4 ± 1.42	0.822	0.408	
Change	−0.20 ± 0.99		−1.41 ± 1.00				0.394
Glucose (mg/dL) *^∮^*	88.3 ± 1.19	89.0 ± 1.27	87.8 ± 0.93	88.9 ± 1.35	0.863	0.955	
Change	0.73 ± 0.85		1.10 ± 1.01				0.783
Triglyceride (mg/dL) *^∮^*	122.6 ± 6.31	126.3 ± 6.25	123.0 ± 8.41	138.0 ± 7.39 **	0.816	0.230	
Change	3.66 ± 5.49		15.0 ± 8.11				0.243
Total cholesterol (mg/dL) *^∮^*	206.7 ± 4.38	204.8 ± 4.35	209.2 ± 4.10	208.3 ± 4.07	0.585	0.488	
Change	−1.86 ± 3.00		−0.93 ± 2.32				0.807
HDL-cholesterol (mg/dL) *^∮^*	54.8 ± 1.54	54.0 ± 1.63	54.8 ± 1.64	54.7 ± 1.71	0.920	0.810	
Change	−0.75 ± 1.15		−0.11 ± 0.88				0.662
LDL-cholesterol (mg/dL) *^∮^*	127.4 ± 4.39	125.6 ± 4.09	129.8 ± 4.02	126.0 ± 3.64	0.529	0.747	
Change	−1.85 ± 2.71		−3.82 ± 2.49				0.595
Serum albumin (mg/dL) *^∮^*	4.55 ± 0.02	4.55 ± 0.03	4.55 ± 0.02	4.52 ± 0.03	0.939	0.497	
Change	−0.01 ± 0.02		−0.03 ± 0.02				0.361
White blood cells (×10^3^/μL) *^∮^*	5.33 ± 0.12	5.29 ± 0.14	5.61 ± 0.13	5.71 ± 0.18	0.089	0.070	
Change	−0.03 ± 0.12		0.10 ± 0.13				0.424
hs-CRP (mg/L) *^∮^*	0.80 ± 0.07	2.01 ± 0.71 *	1.24 ± 0.26	1.77 ± 0.50	0.449	0.781	
Change	1.21 ± 0.72		0.53 ± 0.55				0.460
TNF-α (pg/mL) *^∮^*	22.5 ± 4.93	22.9 ± 5.58	23.1 ± 4.46	21.4 ± 3.13	0.798	0.754	
Change	0.39 ± 2.23		−1.77 ± 2.62				0.529
Immunoglobulin G3 (mg/dL) *^∮^*	265.6 ± 18.1	266.2 ± 18.6	256.8 ± 20.4	246.0 ± 18.5	0.565	0.506	
Change	0.66 ± 14.6		−10.8 ± 11.9				0.548

Mean ± SE. *^∮^* tested by logarithmic transformation, *p^a^*-values derived from independent *t*-tests at baseline. *p^b^*-values derived from independent *t*-tests at follow-up. *p^c^*-values derived from independent *t*-tests at changed value. * p < 0.05 and ** p < 0.01 derived from paired *t*-tests.

**Table 2:** Effects on natural killer (NK) cell activity following 12 weeks of consuming dairy yogurt containing *L. paracasei*, *B. lactis* and heat-treated *L. plantarum*.

	Total Subjects (*n* = 152)				*p^a^*	*p^b^*	*p^c^*	*p^d^*
	Placebo (*n* = 79)		Test (*n* = 73)					
	Baseline	Follow-up	Baseline	Follow-up				
NK cell activity 10:1 (%) *^∮^*	23.9 ± 1.72	26.1 ± 1.79	22.0 ± 1.77	35.1 ± 2.16 ***	0.395	<0.001		
Change	2.26 ± 1.82		13.2 ± 2.00				<0.001	<0.001
NK cell activity 5:1 (%) *^∮^*	17.3 ± 1.34	18.0 ± 1.26	14.6 ± 1.34	25.2 ± 1.87 ***	0.349	0.002		
Change	0.69 ± 1.55		10.6 ± 1.78				<0.001	<0.001
NK cell activity 2.5:1 (%) *^∮^*	12.4 ± 0.94	13.1 ± 1.09	12.0 ± 1.19	20.4 ± 1.54 ***	0.157	<0.001		
Change	0.73 ± 1.22		8.33 ± 1.55				<0.001	<0.001
NK cell activity 1.25:1 (%) *^∮^*	10.5 ± 1.01	11.6 ± 1.15	9.78 ± 1.14	16.9 ± 1.61 ***	0.190	0.001		
Change	1.11 ± 1.32		7.08 ± 1.60				0.004	0.004
NK cell activity 0.625:1 (%) *^∮^*	10.2 ± 1.15	9.57 ± 0.95	8.48 ± 1.20	14.7 ± 1.75 ***	0.059	0.019		
Change	−0.65 ± 1.36		6.23 ± 1.66				0.002	0.002

Mean ± SE. *^∮^* tested by logarithmic transformation, *p^a^*-values derived from independent *t*-tests at baseline. *p^b^*-values derived from independent *t*-tests at follow-up. *p^c^*-values derived from independent *t*-tests at changed value. *p^d^*-values adjusted for baseline values, sex, and changes in diastolic BP for changed value. *** p < 0.001 derived from paired *t*-tests.

## References

[1] Gavazzi G., Krause K.H. (2002). Ageing and infection. Lancet Infect. Dis..

[2] Ben-Yehuda A., Weksler M.E. (1992). Immune senescence: Mechanisms and clinical implications. Cancer Investig..

[3] Makinodan T., James S.J., Inamizu T., Chang M.P. (1984). Immunologic basis for susceptibility to infection in the aged. Gerontology.

[4] Adolfsson O., Meydani S.N., Russell R.M. (2004). Yogurt and gut function. Am. J. Clin. Nutr..

[5] El-Abbadi N.H., Dao M.C., Meydani S.N. (2014). Yogurt: Role in healthy and active aging. Am. J. Clin. Nutr..

[6] Ashraf R., Shah N.P. (2014). Immune system stimulation by probiotic microorganisms. Crit. Rev. Food Sci. Nutr..

[7] Dallal M.M.S., Yazdi M.H., Holakuyee M., Hassan Z.M., Abolhassani M., Mahdavi M. (2012). *Lactobacillus casei* ssp. casei induced Th1 cytokine profile and natural killer cells activity in invasive ductal carcinoma bearing mice. Iran. J. Allergy Asthma Immunol..

[8] Kawashima T., Hayashi K., Kosaka A., Kawashima M., Igarashi T., Tsutsui H., Obata A. (2011). *Lactobacillus plantarum* strain YU from fermented foods activates Th1 and protective immune responses. Int. Immunopharmacol..

[9] Makino S., Ikegami S., Kume A., Horiuchi H., Sasaki H., Orii N. (2010). Reducing the risk of infection in the elderly by dietary intake of yoghurt fermented with *Lactobacillus delbrueckii* ssp. *bulgaricus* OLL1073R-1. Br. J. Nutr..

[10] Rizzardini G., Eskesen D., Calder P.C., Capetti A., Jespersen L., Clerici M. (2012). Evaluation of the immune benefits of two probiotic strains *Bifidobacterium animalis* ssp. *lactis*, BB-12^®^ and *Lactobacillus paracasei* ssp. *paracasei*, L. casei 431^®^ in an influenza vaccination model: A randomised, double-blind, placebo-controlled study. Br. J. Nutr..

[11] Tobita K., Yanaka H., Otani H. (2009). Heat-treated *Lactobacillus crispatus* KT strains reduce allergic symptoms in mice. J. Agric. Food Chem..

[12] Murosaki S., Yamamoto Y., Ito K., Inokuchi T., Kusaka H., Ikeda H., Yoshikai Y. (1998). Heat-killed *Lactobacillus plantarum* L-137 suppresses naturally fed antigen-specific IgE production by stimulation of IL-12 production in mice. J. Allergy Clin. Immunol..

[13] Hirose Y., Murosaki S., Yamamoto Y., Yoshikai Y., Tsuru T. (2006). Daily intake of heat-killed *Lactobacillus plantarum* L-137 augments acquired immunity in healthy adults. J. Nutr..

[14] Lee H.A., Kim H., Lee K.W., Park K.Y. (2015). Dead nano-sized *Lactobacillus plantarum* inhibits azoxymethane/dextran sulfate sodium-induced colon cancer in Balb/c mice. J. Med. Food.

[15] Takeda K., Okumura K. (2007). Effects of a fermented milk drink containing *Lactobacillus casei* strain Shirota on the human NK-cell activity. J. Nutr..

[16] Makino S., Ikegami S., Kano H., Sashihara T., Sugano H., Horiuchi H., Oda M. (2006). Immunomodulatory effects of polysaccharides produced by *Lactobacillus delbrueckii* ssp. *bulgaricus* OLL1073R-1. J. Dairy Sci..

[17] Vidal S.M., Khakoo S.I., Biron C.A. (2011). Natural killer cell responses during viral infections: Flexibility and conditioning of innate immunity by experience. Curr. Opin. Virol..

[18] Ogata K., An E., Shioi Y., Nakamura K., Luo S., Yokose N., Dan K. (2001). Association between natural killer cell activity and infection in immunologically normal elderly people. Clin. Exp. Immunol..

[19] Heremans H., Dillen C., van Damme J., Billiau A. (1994). Essential role for natural killer cells in the lethal lipopolysaccharide-induced Shwartzman-like reaction in mice. Eur. J. Immunol..

[20] Schroder K., Hertzog P.J., Ravasi T., Hume D.A. (2004). Interferon-γ: An overview of signals, mechanisms and functions. J. Leukoc. Biol..

[21] Sayers T.J., Mason L.H., Wiltrout T.A. (1990). Trafficking and activation of murine natural killer cells: Differing roles for IFN-γ and IL-2. Cell. Immunol..

[22] Harris D.P., Haynes L., Sayles P.C., Duso D.K., Eaton S.M., Lepak N.M., Lund F.E. (2000). Reciprocal regulation of polarized cytokine production by effector B and T cells. Nat. Immunol..

[23] Nair M.P., Kandaswami C., Mahajan S., Chadha K.C., Chawda R., Nair H., Schwartz S.A. (2002). The flavonoid, quercetin, differentially regulates Th-1 (IFNγ) and Th-2 (IL4) cytokine gene expression by normal peripheral blood mononuclear cells. Biochim. Biophys. Acta.

[24] Ertel W., Keel M., Neidhardt R., Steckholzer U., Kremer J.P., Ungethuem U., Trentz O. (1997). Inhibition of the defense system stimulating interleukin-12 interferon-γ pathway during critical illness. Blood.

[25] Trinchieri G. (1998). Proinflammatory and immunoregulatory functions of interleukin-12. Int. Rev. Immunol..

[26] Klein-Franke A., Anderer F.A. (1995). IL-12-mediated activation of MHC-unrestricted cytotoxicity of human PBMC subpopulations: Synergic action of a plant rhamnogalacturonan. Anticancer Res..

[27] Kalia V., Sarkar S., Gourley T.S., Rouse B.T., Ahmed R. (2006). Differentiation of memory B and T cells. Curr. Opin. Immunol..

[28] Rimm E.B., Giovannucci E.L., Stampfer M.J., Colditz G.A., Litin L.B., Willett W.C. (1992). Reproducibility and validity of an expanded self-administered semiquantitative food frequency questionnaire among male health professionals. Am. J. Epidemiol..

